# NOVEL METHODS AND DATA IN POPULATION AGING RESEARCH

**DOI:** 10.1093/geroni/igae098.0527

**Published:** 2024-12-31

**Authors:** Ruijia Chen, Eleanor Hayes-Larson

**Affiliations:** Chair; Co-Chair

## Abstract

Causal inference in population health research on aging is critical for understanding determinants of healthy aging and informing potential avenues of intervention. Such research faces a number of methodological challenges including data availability, detection bias, selection bias, confounding, and reverse causation. Novel statistical methods and approaches to leveraging data have been developed to address these issues. This session features cutting-edge research that incorporates novel methods and data to address key methodological challenges in aging research. Speakers will address (1) the use of hypothetical intervention approaches to address confounding and reverse causation to understand the impact of loneliness on memory function in older adults, (2) a multiple-exposure data reduction approach to identify important determinants of exercise in an aging population, (3) ways to uncover and account for detection bias in electronic health record studies of dementia, (4) pooling of cohorts to address gaps in data availability across the lifecourse, and (5) challenges and opportunities related to extending findings from selected aging samples to broader populations of interest. Epidemiology of Aging Interest Group Sponsored Symposium

